# The Sixth-Year Molar

**Published:** 1893-12

**Authors:** P. P. Sparke

**Affiliations:** Richmond, Va.


ARTICLE IV.

### "THE SIXTH-YEAR MOLAR."

THE PRIZE ESSAY AT THE VIRGINIA STATE DENTAL ASSOCIATION.

BY P. P. SPARKE, D. D. S., RICHMOND, VA.

The value of the sixth-year molar is variously regarded by dentists and patients—some esteeming its loss a matter of little concern; some considering its value only as the difference between the cost of extraction and filling, giving preference to that which is cheaper, without a thought of any other; some computing its loss as a mathematical problem, such as one from thirty-two leaves thirty-one, which is no bad showing when compared with the average loss; some believing its value to be associated with many other interests, and, consequently, should be cared for both its own intrinsic worth and other members associated, while there are some fools, who might be denominated ultra-extremists, who carry science and its theories so far that they would at any time destroy the body to save the tooth, the latter unfortunate class being composed almost exclusively of either those who are freshly graduated and have not tested their theories, or those would-be practitioners amongst a questionably cultivated set, who are readily duped by any form of charlatanism which makes a pretense to superiority. Between these two extremes I propose to discuss the six-year molar, regarding its loss, not with that brutal indifference so often observed among the vulgar and ignorant on the one side, and the excessive importance of its preservation, under whatsoever conditions, seen among the foolishly educated and over-sensitive class, on the other.

For my own part, I should advocate the preservation of the sixth-year molar, whether I were able to give a specific reason or not, but would maintain such position upon the general conviction that the Almighty has created no part of our being without the wisdom of some special purpose, and that the function of any one part extends beyond itself and is more or less associated with the whole being.

If I were called upon to point out definitely the functions of the hair, I do not know that I could point out definitely one wise purpose it serves beyond appearance, unless it be to keep the head warm in winter, which advantage is more than offset by keeping it too hot in summer, as some of you in attendance at present can, doubtless, testify. Yet, my conviction is unshaken that be it the hair, the thyroid gland, or some other obscure part, whose functions may or may not be known, the wisdom of its preservation is beyond question.

The study of the sixth-year molar may be made for general information on tooth origin, formation, functions, etc., or for special information as to its own individuality and peculiarities apart from the rest. Believing that the object of the papers requested on this subject is to bring out the distinctive character of this tooth and its points of difference from others, the efforts of this paper shall be toward that end.

The origin of the sixth-year molar, like that of the rest of the teeth, is from the outer covering of the oral cavity, or epithelium, corresponding with the skin outside of this cavity. But, unlike the anterior teeth, the prolongation of epithelium, which sinks into the jaw and forms the sixth-year molar, gives off later a branch which forms the twelfth-year molar, and from the latter yet another branch which forms the so-called wisdom tooth. Thus, it will be seen that while each prolongation of epithelium anterior to the sixth-year molar forms only one tooth that from which this tooth is derived is the source of origin of three, and these three the largest in the arch. This is a peculiarity not associated with the formation of the other teeth to which I would call special attention, desiring to point out some of the results of this peculiarity.

In the first place, the second and third molars in their origin being offshoots from the first, it is very evident that there can be no second or third molars except there be a first. In the anterior teeth one or another will sometimes be found missing without the loss of adjoining ones, but from the very nature of the origin of the sixth-year molar it could not fail to develop without precluding the emption of the second and third. Sometimes it seems as if the powers of formation of this epithelial prolongation gave out before reaching the third molar, as is very often witnessed by the absence of the wisdom tooth. But it is not likely that anyone ever observed a wisdom tooth which had no sixth-year molar as its predecessor. For my own part, I never did, nor can I conceive of the formation of either second or third molars without the first after a study of their origin. Therefore, to be brief, I might say, no sixth-year molar, no molars at all. But there is connected with this tooth another striking feature associated with its origin, namely, absence of irregularity. Though any of the anterior teeth are sometimes found out of position, as may also be the case with the deno sapiential, probably, this tooth was never seen out of its regular position except as the result of violence, and, according to my observation, it has never been out of position except from injury subsequent to its eruption. The absence of its irregularity is readily understood when we recollect that it, having no temporary predecessor to retard its eruption or change its course, and having the posterior part of the jaw to pick its course, invariably comes out in a normal position. The situation also of this tooth makes it the keystone of the arch, its loss in the adult tending more to cause irregularity than the sacrifice of any other. Being in the center of the arch, its anterior surface forming the dividing line on the alveolus between its anterior and posterior halves, it locks the anterior and posterior teeth in position. While, from its location, it is most active in preserving the regularity of the rest of the teeth, its arrangement in that location makes it most active in the process of mastication. This will be understood when we note that in the upper jaw the first molar is the point at which the jaw teeth curve upward and backward, and the front curve upward and forward, while in the lower jaw the first molar is the lowest point in the curve. Thus, when food is placed in the mouth it naturally seeks the lowest point or hollow in the curve, which corresponds with the first molar of the lower jaw, where it is masticated by the upper first molar which points downward farthest in the upper jaw, fitting and crushing the food in such depression. These teeth, therefore, when in their normal position from their arrangement, are forced to do the greater part of mastication, and may be termed the upper and nether millstones of the mouth. The food, chewed upon either the front teeth or second and third molars, can be kept in position and masticated only by more or less effort which is not natural, the position which it naturally takes when left to itself being in the depression in the lower arch over the first molar, as mentioned before, where it may be crushed by its superior antagonist. The preservation of this tooth, therefore, is more to be desired from the standpoint of intrinsic value than any other. Notwithstanding that this is the most valuable of dental organs, there are times when its extraction is indicated, which will be pointed out further on.

The form of this tooth is, also, said to differ from that of the rest of the molars, but as it has no special bearing upon any new thought desired to be brought out in this paper it need not be dwelt upon.

With the single exception of irregularity, the troubles to which this tooth is liable are the same as for the others with the difference that it is more liable to such troubles, and, consequently, its treatment has to be modified with this fact kept in mind. As an example, take caries, to which all the teeth are liable, but none so much so as the sixth-year molar. Evidently, there is some causes why it should decay more readily than any other of the dental organs, and it becomes our duty to search out the causes, so that we may act intelligently in its treatment. In the study of its structure, composition and the changes which it undergoes at different periods of life we may get the key to the problem. It is generally acknowledged that early in life, soon after its eruption, it consists much more largely of animal matter than at a later period, when the earthy material has been deposited in larger quantities, consequently, disorganization, which may be started soon after its eruption, is liable to prove much more destructive than if started later. While it is highly important that this tooth should be preserved for the sake of itself as well as those associated with it, if decay begins very early, say between the ages of six and ten years, and reaches the nerve it is much better to extract at once than attempt at such an early age the destruction of its nerve and root filling. There are several reasons why it is better at this age to extract than fill as a dead tooth. In the first place, at this time the animal matter in the tubuli of crown and roots is in very large proportion and more liable to putrefactive changes than later, when the earthy deposits reduce the size of the tubuli. In the second place, the tissues of the jaw, like other parts of the body, in the young are more susceptible to irritation than later in life. Thus, an abscess might be produced by an irritating body which might readily be tolerated at a later period. In the third place, if, from the condition of this tooth when it comes under observation, it is seen that it can be preserved for only a few years, it would be best to extract immediately, which operation would give the second and third molars an opportunity to come forward during and after eruption, and fill the space caused by the loss of the first. Though it may be best, as a rule, to save the natural teeth for only a few years, if they cannot be saved longer, the rule will not hold good for this, the most valuable of all teeth, at the early age and under the conditions indicated. Patients who present themselves to me between the ages of six and ten years with the six-year molars unfavorable toward permanent salvation I extract. In this manner, such teeth as cannot be saved except for, possibly, a few years are removed from the danger of causing an irregular position of others, which would result if extracted subsequently. To sum it up, I would say that when extraction is unavoidable, it is better to extract at ten than at eighteen years of age. After such an operation the second and third molars will generally fill the space very satisfactorily, which would not be the case if the first molar were extracted about the latter year, with, most likely, an abscess to contend with up to that time. But, where the nerves remain alive, the density of the tooth may be hastened by the use of oxyphosphate filling material in the cavities to be filled in preference to amalgam until such time as the tooth by increasing density from the deposition of lime salts, assisted by the hardening influence of the oxyphosphate material, becomes hard enough for metallic fillings.

It is a fact that has received due recognition that an oxyphosphate filling will either hasten the increase of density of a tooth or increase the density through some virtue of its own.

So far, I have never learned whether the oxyphosphate filling hastens the deposition of lime salts in the tubuli of a tooth or of itself acts upon the tubuli of the dentine in immediate contact. But, whatsoever be the mode of action, it has a most desirable effect in hardening the dentine of a tooth in which it is placed. I often use it, intending it to remain for only a year or two, for the sole purpose of hardening the tooth, so that the metallic filling purposed to be used after that length of time may be employed with greater prospects of permanency. Attention should be called to the first molar as a butt for bridge-work, when the choice lies between it and adjoining roots, for the following reasons:

1st. It is not inclined to change its position in the jaw so readily as the other molars, whose tendency is to work forward or in the front ones to change with slight pressure. 2nd. That it is naturally the point at which the strain in mastication falls, thus becoming the butt proper for work of this kind. As all objects have a center of balance and a point upon which strain falls more heavily than elsewhere, so have the jaws, and this center of strain corresponds with the location of the first molars. For this reason gold crowns for the first molars should be thicker than elsewhere on account of both wear and strain being greater. Very thin gold crowns here are contrary to reason and the necessities of the case.

There are some other observations in connection with the first molar, but as those mentioned are some of its peculiarities in which it differs markedly from the other teeth, and, as the object of this paper is to bring out its distinctive characteristics, I hope that through a discussion by other members we may all gain new ideas on the sixth-year molar.—*Southern Dental Journal.*

---

ARTICLE V.

